# Sense of belonging in veterinary education: A scoping review

**DOI:** 10.1002/vro2.70045

**Published:** 2026-07-20

**Authors:** Claudia A. Rivera Munoz, Laura M. Dooley

**Affiliations:** ^1^ The University of Melbourne Melbourne Victoria Australia; ^2^ Melbourne Veterinary School The University of Melbourne Parkville Victoria Australia

**Keywords:** peer interactions, wellbeing, sense of belonging, inclusion, veterinary education

## Abstract

**Background:**

Students’ sense of belonging is increasingly recognised as an important enabler of student engagement and wellbeing in health profession education. Understanding the role of students’ sense of belonging in veterinary education may inform targeted interventions and guide further research in the discipline. This scoping review examined how belonging has been conceptualised in veterinary education literature and examined reported factors and outcomes associated with students’ sense of belonging.

**Methods:**

A search was conducted across 11 databases, screening 1572 records. Studies were eligible if they addressed students’ sense of belonging (or closely related constructs) in veterinary education and reported factors or outcomes. Eight studies met inclusion criteria and were synthesised thematically.

**Results:**

The analysis revealed the limited conceptualisation of belonging in veterinary education. Peer interactions can encourage students’ sense of belonging, constituting a key enabler of sense of belonging. By contrast, lack of diversity in the veterinary programme, and experiences of discrimination and racism appeared to hinder students’ sense of belonging, especially for diverse students. Academic performance and satisfaction were also associated with belonging.

**Conclusions:**

Current evidence is limited and conceptually inconsistent. While fostering peer connections and inclusive educational cultures appear to be promising enablers of belonging in veterinary education, further research is needed to more robustly evaluate potential interventions.

## INTRODUCTION

### Students’ sense of belonging

A sense of belonging has emerged as a key contributor to student academic motivation,^1^ engagement,^2^, ^3^ success,^4^ persistence in studies^5^ and wellbeing^6^, ^7^ across higher education. In this context, students’ sense of belonging can be defined as ‘students perceived social support on campus, a feeling or sensation of connectedness, and the experience of mattering or feeling cared about, accepted, respected, valued by, and important to the campus community or others on campus such as faculty, staff and peers’ (p4).^8^ While there is limited agreement in its conceptualisation,^4^, ^9^ sense of belonging has been described as a complex and multidimensional construct that evolves over time and is influenced by multiple social and contextual factors,^10^, ^11^ including a positive overall student experience,^12^ inclusive classroom environments and teaching practices,^4^ and supportive interactions with peers^4^, ^13^ and educators.^4^, ^14^

In terms of multidimensionality, sense of belonging has been associated with academic, social, spatial or place‐based and personal dimensions. The academic dimension often refers to the feeling of fit in the course or programme and students’ feelings that they are in the right place.^15^, ^16^ Students’ academic belonging can encourage students’ self‐efficacy, participation in classes and developing career identity, especially when students’ learning experiences are perceived to contribute to their future career.^4^, ^15^, ^16^ This dimension can also involve feeling part of a group or sense of membership,^5^, ^15^ including being part of a community or team within the programme^17^ (e.g., community of practice in medicine). The social dimension refers to the interpersonal relationships and peer networks that contribute to a student's sense of connectedness within the learning community, including friendships and peer support.^15^ The spatial dimension refers to feelings of belonging to a place in geographical and cultural sense, for example, a sense of belonging to the university campus or cultural identity of the place.^15^ The personal dimension refers to students’ identity, life satisfaction and personal interests associated with a sense of belonging.^15^

### Students’ sense of belonging in health profession education

Across health profession education, there has been limited conceptualisation of students’ sense of belonging.^9^ In nursing education, belongingness has been conceptualised as ‘a deeply personal and contextually mediated experience that evolves in response to the degree to which an individual feels (a) secure, accepted, included, valued and respected by a defined group, (b) connected with or integral to the group, and (c) that their professional and/or personal values are in harmony with those of the group’ (p2872).^18^ In medical education, Quaintance et al. defined belonging as ‘as feeling part of the group, team or profession, fitting in interpersonally and/or professionally without pretending’ (p2036).^19^ Both definitions highlight how professional and personal values in relation to the group contribute to a sense of belonging in professional learning contexts.

In health profession education, a sense of belonging has been associated with preparedness for clinical practice, academic persistence^7^ and professional identity formation.^17^, ^19^ In medical education, scholars have suggested that the experience of belonging or exclusion to the profession influences professional identity formation. Learning environments that foster belonging to the profession provide clear expectations and norms about being a professional, support and value students’ contributions in the clinical setting, and offer multiple opportunities for interpersonal connections between peers and educators.^19^ However, students from diverse backgrounds tend to report difficulties with their sense of belonging to the profession, risking their professional identity formation. For example, women and students from underrepresented groups (e.g., ethnic minority backgrounds, first‐generation, low socioeconomic background students) tend to report discrimination and isolation in medical programmes that can impact their feelings of belonging to the profession.^19^, ^20^

Veterinary education represents a distinctive educational context in which belonging can be considered particularly pertinent for several interconnected reasons. First, veterinary programmes involve a process of professional socialisation through a variety of pre‐clinical and work‐integrated learning contexts.^21^ Students must navigate transitions across multiple and heterogenous learning environments that deliver their theoretical, practical and professional learning experiences. Second, across these environments they experience a great diversity of veterinary professional roles, groups and identities. Furthermore, alongside this learning, they are developing their own sense of individual values, purpose and identity.^21^ Belonging in veterinary education is likely to be multidimensional with individuals experiencing belonging to peer groups, institutions, disciplines of veterinary science, clinical teams and to the wider profession. In this way, students’ sense of belonging can play a crucial role in shaping their overall learning experiences and professional aspirations and trajectories.

Exploring students’ sense of belonging in veterinary education may be crucial, considering the concerning mental health and wellbeing evidence. Veterinary students experience high levels of psychological distress and poorer wellbeing^22^ when compared with similar student populations. Studies^23^, ^24^ have indicated that veterinary students reported high levels of stress associated with high academic demands and expectations in the course and concerns about their academic performance. In this setting, belonging could be a protective factor^25^ that supports student wellbeing and resilience through the challenges of their training and professional lives.

Diversity and inclusion are also emerging concerns for veterinary education and other health professions. For example, studies in medical education^20^ have suggested that students’ experiences of belonging differ across students from diverse social and educational backgrounds, who often report difficulties to belonging to the university and profession. With increasingly diverse student populations, there has been a growing emphasis on student wellbeing, diversity and inclusion in veterinary education^26^ and understanding the factors contributing to belonging has become a priority for educators. In this context, it is timely to critically examine the existing literature about belonging in veterinary education.

While belonging has been extensively studied in higher education and, to some extent, in medical^20^ and nursing education,^7^ the veterinary education literature remains comparatively fragmented, with no currently published scoping or systematic reviews. This scoping review addresses this gap by exploring conceptualisation, factors and outcomes of students’ sense of belonging in veterinary education literature. An understanding of the current evidence can inform future research and educational practice aimed at fostering belonging, wellbeing and inclusion in veterinary education. These educational practices may shape graduates’ sense of belonging and their capacity for sustained engagement in the veterinary profession, a particularly pertinent outcome in the current context of widespread workforce shortages.

### Aim and research questions

The present scoping review aimed to evaluate how students’ sense of belonging in veterinary education has been reported in the literature, focusing on how researchers have conceptualised a sense of belonging in higher education and reported factors and outcomes influencing veterinary students’ sense of belonging. Three research questions guided this scoping review:

RQ1. How is students’ sense of belonging (or lack of belonging) in veterinary education currently conceptualised in the higher education literature?

RQ2. What factors are associated with veterinary students’ sense of belonging?

RQ3. What outcomes are associated with veterinary students’ sense of belonging?

## METHODS

The scoping review followed the Arksey and O'Malley's five‐stage framework.^27^ Once the protocol and research questions were developed (stage 1), the authors determined the search terms and conducted initial searches between July 2025 and August 2025 (stage 2). The search terms are presented in Table 1, considering the setting, student population, concept and factors and outcomes.

**TABLE 1:** Search terms used in the scoping review.

Category	Search terms
Setting	Veterinary or veterinary education
Student population	undergraduate; bachelor's degree; postgraduate; post‐graduate; graduate coursework; capstone; masters; first year; freshmen
Concept	‘sense of belonging’; belong*
Factor or outcome	factor*; concept*; understand*; defin*; perception; operationali*; experience; influen*; contribut*; determinant; predict*; precond*; barrier; enabl*; effect; condition; correlate; precondition; outcome; intervention; impact; consequen*; predisposition; obstacle; impediment; driver

The initial searches were conducted in 11 databases by the first author: Proquest Central; Ebscohost: Educational Resources Information Center and Academic Search Complete; PubMed; Web of Science; Medline; Scopus; PsycINFO; Embase; CAB Abstracts; CINAHL; and Google Scholar. When available in the database, the setting and concept were restricted to the title/abstract or subject level to strengthen the relevance of the literature. Table 2 presents examples of first and second searches in the PubMed database.

**TABLE 2:** Examples of search string used in the first and second searches in PubMed.

Search	Search string
First search	(‘sense of belonging’ [Title/Abstract] OR belong*[Title/Abstract]) AND (veterinary[Title/Abstract]) AND (undergraduate OR bachelor's degree OR postgraduate OR post‐graduate OR graduate OR coursework OR capstone OR masters OR first year OR freshmen OR student) AND (higher education OR university OR college) AND (factor* OR concept* OR understand* OR defin* OR perception OR operationali* OR experience OR influen* OR contribut* OR determinant OR predict* OR precond* OR barrier OR enabl* OR effect OR condition OR correlate OR precondition OR outcome OR intervention OR impact OR consequen* OR predisposition OR obstacle OR impediment OR driver)
Second search	(Connect*[Title/Abstract] OR Member*[Title/Abstract] OR Communit*[Title/Abstract] OR Lonel*[Title/Abstract] OR Affiliat*[Title/Abstract] OR Inclus*[Title/Abstract]) AND (undergraduate OR bachelor's degree OR postgraduate OR post‐graduate OR graduate OR coursework OR capstone OR masters OR first year OR freshmen OR student) AND (higher education OR university OR college) AND (factor* OR concept* OR understand* OR defin* OR perception OR operationali* OR experience OR influen* OR contribut* OR determinant OR predict* OR precond* OR barrier OR enabl* OR effect OR condition OR correlate OR precondition OR outcome OR intervention OR impact OR consequen* OR predisposition OR obstacle OR impediment OR driver)

In stage 3, the studies were selected based on the following inclusion criteria: (a) studies published between 1 January 2010 and 30 June 2025; (b) English‐language studies; (c) peer‐reviewed articles, PhD and Masters’ dissertations, and grey literature (e.g., reports); and (d) conceptual and empirical studies, including all methodologies. Studies published from 1 January 2010 were included, as this date precedes the rapid growth of the belonging literature in higher education following the publication of influential frameworks in this period and ensures that the review captures contemporary educational contexts and discourse relevant to veterinary education. After completing the initial search, a limited number of studies matched the selection criteria. The authors decided to include broader terms that have been associated with sense of belonging in previous studies^4^ such as connectedness, affiliation and membership. The additional search terms were: Connect*, Member*, Communit*, Lonel*, Affiliat* and Inclus* (Table 2). This second search was conducted across the 11 databases, using the same inclusion criteria. Only three studies from this search were included in the final sample (*n* = 12) that were later removed from the analysis (please see more detail in stage 5 of the analysis).

Once all the searches were conducted and the results exported, they were uploaded to Covidence, a literature review management programme (www.covidence.org/) that was used to manage the screening and data extraction stages. All abstracts were double screened independently by the two authors, excluding literature that did not define or conceptualise belonging in higher education for veterinary students or did not collect data relating to an influencing factor or outcome of belonging in higher education for veterinary students. When there were disagreements in the selection of studies, the authors discussed them and reached a consensus about the study. Both authors also conducted the full‐text screening of the literature, considering the following additional exclusion criteria:

- Wrong population: empirical or conceptual studies that were not based on veterinary students or included veterinary students as one of the study groups but did not provide substantial discussion of veterinary students.
- Studies did not discuss belonging in higher education or the related concept (e.g., sense of connectedness, sense of community, affiliation) in relation to students’ university experience and/or students learning at university.

**FIGURE 1:**
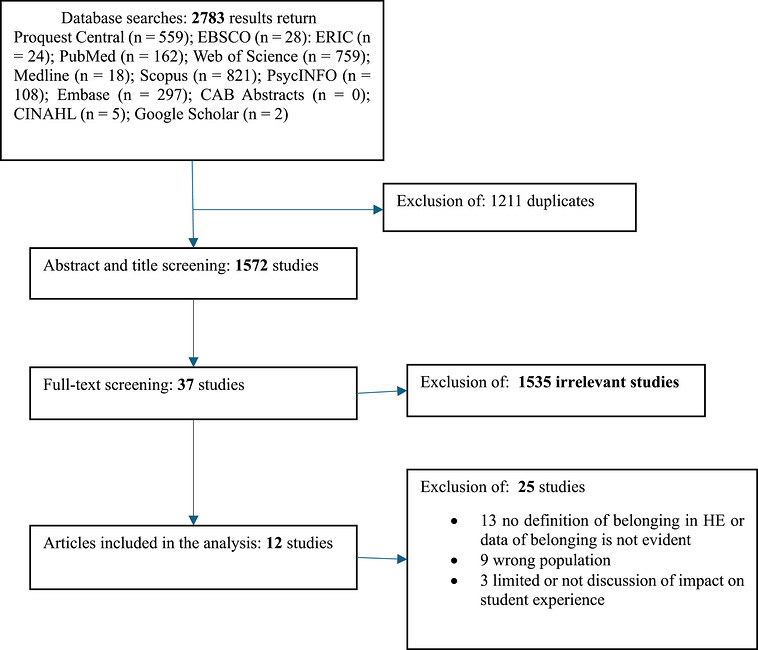
PRISMA (The Preferred Reporting Items for Systematic Reviews and Meta‐Analyses) diagram of selection of articles.

In stage 4, the final pool of studies was identified (Figure 1). The authors used a Microsoft Excel (Microsoft 365, Microsoft Corporation) spreadsheet to record important information about each study, including author(s), year of publication, study location (country/ies), study population (veterinary nursing/veterinary medicine), study aims, method, definition/conceptualisation of belonging and citation (if provided), influencing factor(s) and outcome(s) of belonging. The extraction of information was conducted by the first author and revised by the second author. To address RQ1 (conceptualisation of belonging), we extracted any explicit definitions, conceptual statements, or theoretical frameworks cited in relation to belonging from each included study. These were coded and compared across studies to map the degree of conceptual consistency and the theoretical sources drawn upon by veterinary education researchers. To address RQ2 and RQ3, the authors defined factors as those variables that appeared to influence students’ sense of belonging, enabling or hindering (e.g., peer interactions) based on what was reported in the articles. Outcomes were conceptualised as those variables that appeared to be a consequence of, or a result of sense of belonging (e.g., mental health) based on what was reported in the studies. While we acknowledge that some variables may be both, factors and outcomes, we distinguished them for analysis purposes based on how it was reported in the studies. The factors and outcomes were thematically analysed, by identifying common themes across the reported factors and outcomes, such as peer connections. This analysis was led by the first author and revised by the second author. Both authors discussed and agreed on the themes before writing the results.

Finally, in stage 5, both authors conducted descriptive analysis of the 12 studies selected to summarise their characteristics (e.g., country and type of methodology). Of the 12 studies analysed, eight examined students’ sense of belonging. The other four studies evaluated college climate,^28^ gender discrimination,^29^ social integration^30^ and wellbeing.^31^ While these topics are related to sense of belonging, the studies presented limited discussion of sense of belonging in the higher education context. After further revision, we decided to remove these four studies from the analysis. The findings section focused then on the eight studies that explored students’ sense of belonging in veterinary medical education and veterinary nursing (Table 3). Of the eight studies included in the analysis, three studies described factors and outcomes associated with belonging, four studies mentioned factors only, and one study included outcomes only. The thematic analysis produced four overarching themes, which are used to structure the findings section.

**TABLE 3:** Characteristics of eight studies included in the scoping review.

Authors	Year	Country	Aim	Study design	Sense of belonging in higher education		
					Definition	Factor	Outcome
Burkhard MJ, Dawkins S, Knoblaugh SE, El‐Khoury C, Coble D, Malbrue RA, et al.^34^	2022	United States	Share practices and initiatives to enhance diversity, equity, inclusion and belonging in a veterinary school	Opinion article	✓		
Cardwell JM, Lewis EG^37^	2017	UK	Explored veterinary students’ perspectives and experiences of student life to understand their general wellbeing	Interview study		✓	✓
Chung GH, Armitage‐Chan E^33^	2022	UK	Examined the experiences of Black, Asian or minority ethnic students at a predominantly White institution	Focus group study	✓	✓	
Holt SL, Farrell M, Corrigan RH^40^	2024	UK	Examined veterinary nursing students’ satisfaction within the clinical learning environment and factors influencing their satisfaction	Cross‐sectional survey study		✓	
Holt SL, Mason J, Farrell M, Corrigan RH, Warman S^41^	2024	UK	Investigated student veterinary nurses’ sociocultural experiences in the training veterinary practice and their professional development	Interview study			✓
King N, Cardwell JM, Lewis EG, Patel‐Cook NR, Thuranira‐McKeever C, Crossley VJ, et al.^35^	2025	UK	Examined the psychological effects of racism on Black, Asian and minority ethnic veterinary professionals and students	Cross‐sectional survey study	✓	✓	✓
Little WB, Hervé‐Claude LP, French H, Bradtke J, Artemiou E^32^	2023	United States	Evaluated first‐year veterinary students' belonging and accountability and its link with academic success within curricular change in a subject	Cross‐sectional survey study	✓	✓	
Sample SH, Artemiou E, Donszelmann DJ, Adams C^25^	2025	Canada	Examined veterinary students' experiences of stress, resiliency and mental health awareness initiatives, in relation to the objective structured clinical examination assessment	Interview study		✓	

*Note*: All the articles were peer reviewed.

### Findings

Table 4 presents the characteristics of the eight studies included in the analysis. Half of the studies (*n* = 4) were published between 2023 and 2025 and used qualitative approaches, including interviews (*n* = 3) and focus groups (*n* = 1).

**TABLE 4:** Characteristics of studies analysed (*n* = 8).

Characteristics	*n*
Year	
2015‒2018	2
2019‒2022	2
2023‒2025	4
Country	
UK	5
United States	2
Canada	1
Study method	
Interview or focus group study	4
Survey study	3
Practice report	1
Study population	
Veterinary medical students	6
Veterinary nursing students	2

The findings are organised around four themes generated through the thematic analysis: (1) belonging as feeling included in and connected to a group; (2) connection to peers and educators as associated with student belonging; (3) experiencing discrimination as hindering student belonging; and (4) sense of belonging influencing students’ academic confidence. Conceptualisations of belonging (RQ1) are addressed primarily in theme 1, factors associated with belonging (RQ2) are addressed in themes 2, 3 and 4, and outcomes of belonging (RQ3) are addressed within themes 3 and 4.

### Belonging as feeling included in and connected to a group

There was limited conceptualisation of sense of belonging in the eight studies revised. Only four^32^, ^33^, ^34^, ^35^ provided a definition of sense of belonging in higher education. The four studies provided a conceptualisation of belonging as feelings of membership or connectedness to a specific group or context, including academic setting,^32^ and social or professional context,^33^ in which individuals felt included and accepted.^34^, ^35^ The sources and references cited in the definitions varied across the four studies. While Little et al.^32^ used conceptualisations based on the school context,^36^ Chung and Armitage‐Chan^33^ defined belonging by referencing a study conducted in the veterinary education context included in this review.^37^ King et al.^35^ defined belonging as one of the principles within the identity process theory^38^ to construct and maintain a positive sense of identity. Finally, Burkhard et al.^34^ conceptualised belonging through the lens of diversity, equity and inclusion,^39^ focusing on the individual's perception of feeling welcome and accepted in a particular context.

### Connection to peers and educators is associated with student belonging

Social interactions were one of the most frequent factors associated with students’ sense of belonging, particularly, peer‒peer interactions^25^, ^32^, ^37^ and teacher‒student interactions.^32^, ^37^ Three studies^25^, ^32^, ^37^ suggested that supportive peer connections formed in and outside classes enabled students’ sense of belonging. These supportive peer connections were facilitated when students shared common goals, including career goals such as becoming a veterinarian^25^ and when they participated in collaborative academic environments that promoted working with peers in classes^32^ and supporting each other in their studies.^37^ By contrast, students’ experiences of competitiveness and less supportive classroom environments encouraged feeling uncomfortable with their peers, hindering sense of belonging to class cohort.^37^ Initial social interactions in university events and accommodations in the first year also enabled students’ sense of belonging to the university and increased the likelihood of forming closed friendships (established social connections that are closed to new members) in senior years.^37^ Failing to form these initial connections with peers was perceived as hindering sense of belonging.^37^

Teacher‒student interactions also appeared to enhance students’ sense of belonging, although these were less thoroughly explored in the studies. For example, Little et al.^32^ reported that veterinary students who interacted with educators in classes and actively participated in discussions, tended to feel supported and valued in the classroom, enhancing their sense of belonging. Cardwell and Lewis's study^37^ in the context of clinical placements suggests that positive interactions with senior clinicians also appeared to benefit students’ feeling supported in their learning and facilitating a smooth transition to the profession, enhancing their sense of belonging to the profession.

### Experiencing discrimination hinders student belonging

Two studies discussed the impact of students’ perceptions of lack of diversity and discrimination in academic settings, including classrooms and placements, on their sense of belonging. Chung and Armitage‐Chan^33^ reported how the lack of diversity among students and staff encouraged feelings of ‘otherness’ among students, which tended to hinder students’ sense of belonging. Black, Asian and minority ethnic students also indicated that they experienced difficulties finding an educator from their own ethnicity to ask for support and guidance and that the lack of diverse role models in their institution made them question their career aspirations.^33^ Similarly, King et al.^35^ reported that Black, Asian and minority ethnic students’ feeling like an ‘outsider’ or ‘not welcomed’ in their school and the wider veterinary profession hindered their sense of belonging. In this study, participants mentioned that white students, placement supervisors and lecturers described students from diverse backgrounds (i.e., Black, Asian and other minority groups) as ‘different’ due to their ethnicity, impacting negatively the way students saw themselves within the profession.^35^ Experiences of racism at university (e.g., microinvalidations) also appeared to hinder students’ sense of belonging in the cohort and profession, by reenforcing the perception of being different.^33^, ^35^

### Sense of belonging influencing students’ academic confidence

Two studies^37^, ^40^ discussed academic‐related factors, including academic performance and student satisfaction associated with students’ sense of belonging. Cardwell and Lewis^37^ suggested that veterinary medical students’ poor academic performance can hinder students’ academic sense of belonging. When students received lower grades than expected and perceived that they were at risk of failing, this experience negatively influenced their perceptions of fitting‐in in the course.^37^ Feeling satisfied with the academic experience^40^ can also influence students’ sense of belonging. Holt et al.^40^ found that when veterinary nursing students reported high levels of satisfaction with the clinical learning environment (e.g., having supportive supervisors), they tended to report a higher sense of belonging to the clinical setting.

When compared to studies that examined factors associated with sense of belonging, fewer studies^35^, ^37^, ^41^ reported potential outcomes of sense of belonging. Two studies^35^, ^37^ suggested that students’ sense of belonging influenced their confidence in succeeding in the programme, their participation and abilities to seek help. Holt et al.^41^ reported that veterinary nursing students who felt a sense of belonging to the clinical setting (e.g., felt part of the team), they were more likely to seek help, participate and feel confident in their abilities to perform well in the programme. By contrast, Cardwell and Lewis^37^ reported veterinary medical students’ difficulties developing a sense of belonging to the cohort and described how educational experiences could hinder students’ confidence in their abilities to succeed. Students reported difficulties understanding and meeting the academic requirements at the start of the veterinary programme that led to challenges in developing academic belonging.^37^

## DISCUSSION

This scoping review identified four major themes in the current literature examining sense of belonging in veterinary education: the limited conceptualisation of belonging, the role of social connections, experiences of exclusion and the relationship between academic performance and sense of belonging. While belonging has been extensively studied in general higher education contexts, our findings reveal that the veterinary education literature remains limited in its explicit investigation and conceptualisation of this construct, coinciding with previous literature on sense of belonging.^42^

The observed variability in conceptualisation and definition used across the included studies suggests that veterinary education researchers have not yet established a consensus understanding of this construct. One study^33^ drew on veterinary‐specific literature in conceptualising belonging, suggesting some emerging recognition of the potential impact of context‐specific factors. Current reviews on sense of belonging^10^ suggest the context‐dependent nature of sense of belonging in higher education. Therefore, further research on the role of identity‐based frameworks and diversity, equity and inclusion perspectives will enable the development of a more sophisticated understanding of belonging within the veterinary educational context, grounded in established theoretical foundations of belonging, while acknowledging this unique professional context.

Social interactions were one of the key factors associated with students’ sense of belonging. This review found that having supportive and collaborative peer‒peer interactions can enhance students’ feelings of inclusion and connectedness in academic and professional learning spaces, consistent with previous literature^15^, ^16^ of belonging and the relevance of social connectedness. This finding has distinct pedagogical implications, suggesting that highly interactive and collaborative learning environments (for example, problem‐based learning, peer‐based learning and assessment tasks) can support student learning and foster student belonging,^11^ as suggested in science, technology, engineering and mathematics programmes.^43^ Teacher‒student interactions also demonstrated positive associations with belonging, although these were less examined in the studies reviewed. These findings illustrate that both pedagogical approaches and institutional culture can influence veterinary students’ sense of belonging. Given the collaborative nature of veterinary professional work and the importance of collegial relationships in these working environments, an emphasis fostering social connectedness and collaboration during veterinary education is particularly pertinent.

This review also highlighted the impact of perceived lack of diversity and discrimination on students’ sense of belonging. A feeling of ‘otherness’, experienced particularly by ethnically diverse students, had a negative impact on sense of belonging to the cohort and profession. This finding is similar to studies of underrepresented medical students who tended to report feelings of discomfort, loneliness and isolation in their universities.^20^ Early experiences in classrooms and on placements can shape expectations and insights into the profession, and therefore, observing a lack of diversity within the cohorts can negatively impact students’ views of their future career and their perception of fitting‐in in the profession. This indicates that representation and role‐modelling matters for belonging. When students do not see themselves reflected in peers, educators and the broader profession, this contributes to a feeling that they do not belong. Therefore, while supportive learning environments can be beneficial for students’ sense of belonging, there are systemic and institutional dimensions associated to student cohort and profession that can hinder students’ sense of belonging and potentially impact the formation of a professional identity. Current scholarly discussions about diversity, inclusion and belonging in veterinary education^26^ have started to address these systemic dimensions. However, further work is needed to understand the role of systemic and institutional factors on veterinary students’ sense of belonging.

Another finding is the limited discussion of potential outcomes of students’ sense of belonging in the studies analysed. The discussion of outcomes focuses on how students’ experiencing difficulties with their sense of belonging to the academic dimension of university can hinder students’ confidence in their abilities to succeed in the course. Previous studies in higher education^16^ have suggested that students’ struggling to cope with the academic demands of the programme can impact negatively their academic belonging or the feeling that they are in the right course. This can influence their self‐confidence and self‐efficacy and could lead to disengagement with their studies.^3^ This finding can be concerning considering the high academic expectations and demands in veterinary education, suggesting the relevance of establishing students’ support programmes targeting first‐year transition and teaching strategies with clear academic expectations.

This scoping review provides insights into factors influencing students’ sense of belonging in veterinary education. However, the review has some limitations, suggesting caution in interpreting the findings. The evidence base was small (*n* = 8), limiting the generalisability of the analysis. The included literature was dominated by studies in the UK and included only English‐language sources, which may limit transferability to other contexts. Further research could include literature from non‐English speaking contexts in veterinary education that could offer insights, for example, into experiences of inclusion and exclusion in the veterinary profession. Also, while the search terms used in this review have been adopted in previous scoping reviews on belonging, changes made to the search terms during the review and the exclusion of closely related studies (e.g., those addressing college climate) may have affected identified themes.

This scoping review highlights three areas for further research in advancing the understanding of belonging in the discipline. Further research could focus on developing conceptualisations of students’ sense of belonging from students’ perspectives and their experiences at university, as explored in other disciplines such as nursing.^18^ Considering the current scholarly discussion of diversity and inclusion in veterinary education, further research may explore sense of belonging of diverse students and the academic, contextual and social factors associated to their experiences, as suggested in current studies.^10^ Finally, further empirical studies could explore potential academic and professional outcomes of sense of belonging, including professional identity formation and wellbeing that had been examined in other health profession contexts.^19^ These studies could provide evidence to support practical interventions in the veterinary education context.

## CONCLUSION

This scoping review highlights that collegial peer relationships, supportive educator‐student relationships and visible diversity are important enablers of veterinary students’ sense of belonging, whereas experiences of exclusion and discrimination undermine it. Veterinary educators and institutions should prioritise collaborative pedagogies that facilitate peer interactions and diverse role modelling to foster belonging. However, the evidence is limited, and belonging has been inconsistently conceptualised in the veterinary education literature. Future research is needed to more thoroughly conceptualise belonging in this educational context, and to robustly evaluate the impact of potential interventions.

## AUTHOR CONTRIBUTIONS

Laura M. Dooley: conceptualisation (lead) of the project, methodology (protocol), data analysis, writing revision of manuscript. Claudia A. Rivera Munoz: methodogy (protocol and searches), data analysis, writing of original draft and review and editing of final manuscript.

## CONFLICTS OF INTEREST

The authors declare they have no conflicts of interest.

## FUNDING INFORMATION

The authors received no specific funding for this work.

## ETHICS STATEMENT

The authors confirm that the ethical policies of the journal, as noted on the journal's author guidelines page, have been adhered to. No ethical approval was formally required because this was a review article with no original research data.

## Data Availability

The data used in this scoping review is openly available.
